# Fresh Evidence for Platelets as Neuronal and Innate Immune Cells: Their Role in the Activation, Differentiation, and Deactivation of Th1, Th17, and Tregs during Tissue Inflammation

**DOI:** 10.3389/fimmu.2018.00406

**Published:** 2018-03-02

**Authors:** Eugene D. Ponomarev

**Affiliations:** ^1^Faculty of Medicine, School of Biomedical Sciences, The Chinese University of Hong Kong, Shatin, Hong Kong

**Keywords:** platelets, inflammation, CD4 T cells, glycolipids, damage-associated molecular pattern, neurotransmitter, autoimmunity

## Abstract

Recent studies suggest that in addition to their common function in the regulation of thrombosis and hemostasis, platelets also contribute to tissue inflammation affecting adaptive immunity. Platelets have a number of pro-inflammatory and regulatory mediators stored in their α-granules and dense granules, which are promptly released at sites of inflammation or tissue injury. Platelet-derived mediators include cytokines (IL-1α, IL-1β, and TGFβ1), chemokines (CXCL4 and CCL3), immunomodulatory neurotransmitters (serotonin, dopamine, epinephrine, histamine, and GABA), and other low-molecular-weight mediators. In addition, activated platelets synthesize a number of lipid pro-inflammatory mediators such as platelet-activating factor and prostaglandins/thromboxanes. Notably, platelets express multiple toll-like receptors and MHC class I on their surface and store IgG in their α-granules. Platelet-derived factors are highly effective in directly or indirectly modulating the priming and effector function of various subsets of T cells. Besides secreting soluble factors, activated platelets upregulate a number of integrins, adhesion molecules, and lectins, leading to the formation of platelet–T cells aggregates. Activated platelets are able to instantly release neurotransmitters acting similar to neuronal presynaptic terminals, affecting CD4 T cells and other cells in close contact with them. The formation of platelet–T cell aggregates modulates the functions of T cells *via* direct cell–cell contact interactions and the local release of soluble factors including neurotransmitters. New data suggest an important role for platelets as neuronal and innate-like cells that directly recognize damage- or pathogen- associated molecular patterns and instantly communicate with T cells.

## Introduction

Platelets are small non-nucleated cells around 2–3 μm in diameter, produced by megakaryocytes in the bone marrow by a budding process (1). Platelets have developed a secretory machinery with multiple storage vesicles, mitochondria, mRNA, ribosomes, signal transduction pathways, and multiple receptors acting in many instances as large nucleated cells (2). Most importantly, in the peripheral blood, platelets outnumber mononuclear cells by almost 100-fold and constitute the most abundant population of circulating cells after erythrocytes (1, 2). Platelets are known as a cell type that immediately reacts to damage to the blood vessels and participates in blood clot formation, but their role in the regulation of immune cells such as CD4 T cells is still largely underestimated (1–4).

**Figure 1:**
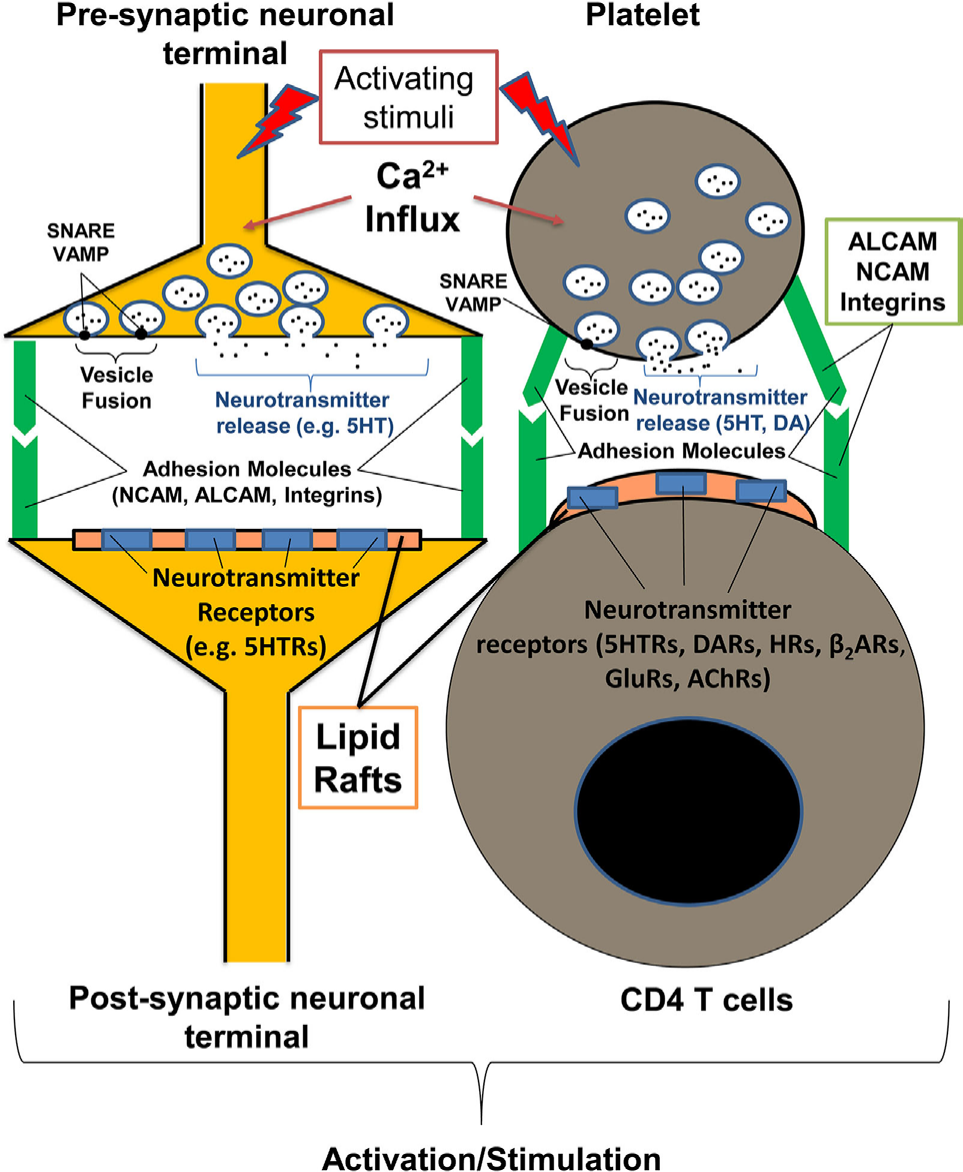
Communication of platelets with CD4 T cells has many similarities with the interaction of presynaptic and postsynaptic neurons. The process of platelet degranulation is very similar to the process of the release of neurotransmitters by presynaptic neurons. In both presynaptic neurons and platelets, neurotransmitters (e.g., serotonin, dopamine), and other mediators are stored in specific vesicles inside the cells. During the process of neuronal or platelet activation, specific vesicles are fused with the surface membrane (using the same docking molecules for platelets and neurons such as VAMP and SNARE), and the vesicle content is released. Both CD4 T cells and postsynaptic neurons have detergent-resistant membrane domains (lipid rafts) with neurotransmitter receptors (e.g., serotonin, dopamine receptors) that promote the further activation of postsynaptic neuron or T cells when stimulated. Both neuronal and platelet–T cell synapses are stabilized with adhesion molecules such as ALCAM, NCAM, and various integrins. ACLAM adhesion molecules and integrins are expressed by neurons, platelets, and activated T cells, and NCAM is expressed by neurons and subsets of activated T cells. During inflammation, platelets are able to directly interact with postsynaptic neurons or activate T cells recognizing specific glycolipids (sialylated gangliosides) and glycoproteins (ALCAM, NCAM) within lipid rafts *via* specific receptors (CD62P, Siglecs, CLRs). AChRs, acetylcholine receptors; CLRs, C-type lectin receptors; DA, dopamine; DARs, dopamine receptors; GluRs, glutamate receptors; HRs, histamine receptors; β_2_ARs, β2-adrenoreceptors; 5HT, serotonin; 5HTRs, serotonin receptors.

Recent research suggests that platelets participate in inflammation by producing a number of pro-inflammatory mediators (1, 5). Platelets store many mediators in their vesicles (granules), which are swiftly released in a manner similar to the release of neurotransmitters from neuronal presynaptic synapses (2, 6, 7). The molecular mechanisms of the calcium-dependent activation of the secretory machinery and the fusion of vesicles with plasma membrane *via* specific docking molecules (e.g., SNAREs, VAMPs, Syntaxins) are very similar for platelets and neuronal cells and aim to release a number of neurotransmitters from platelets with the most abundant monoamine serotonin, followed by the other biogenic amines epinephrine, dopamine, and histamine (6, 8–11). Platelets also have inhibitory neurotransmitter GABA, but at lower concentrations than in biogenic amines (12). Similar to postsynaptic neurons, immune cells, including CD4 T cells, have multiple receptors for neurotransmitters (e.g., serotonin, dopamine receptors), which provide a direct path by which platelets can instantly communicate with CD4 T cells (13, 14). Similar to neuronal synapses, platelets and T cells are capable of making direct contact with other cells such as antigen-presenting cells (immunological synapses) *via* a number of specific adhesion molecules and integrins (1, 15–17). Certain adhesion molecules (e.g., NCAM or CD56) are expressed in both neurons and subsets of activated T cells, while other adhesion molecules (e.g., ALCAM or CD166) are expressed in neuronal cells, T cells, and platelets, and have a high level of structural homology with NCAM (17–21) (Figure 1).

Besides platelet-derived neurotransmitters (serotonin, dopamine, epinephrine, histamine, and GABA), there are other mediators that are either released as soluble factors or appear on the plasma membrane of activated platelets as receptors that directly affect CD4 T cells. These factors include cytokines, chemokines, and potent lipid mediators such as platelet-activating factor (PAF) and thromboxane A2 (2, 22). Activated platelets also release IgGs, which are stored in their α-granules (23). Finally, platelets have a large number of integrins, adhesion molecules, and lectins, which are located inside the granules and are recruited to the platelet plasma membrane when the granules fuse with the plasma membrane (e.g., CD62P) (Table 1) (6, 15, 16). Adhesion molecules play an important role in the formation of platelet–T cell contacts, in a manner similar to the formation of neuronal synapses (17) (Figure 1). Although it is known that platelets release multiple soluble factors and upregulate multiple integrins and adhesion molecules during their activation, it is still not clear which activating stimuli are responsible for the release of proper factor and/or proper surface receptor. It is also not clear how specific is the action of single platelet-derived factor on the proliferation and differentiation of various subsets of CD4 T cells. In an attempt to resolve these questions, we take the opportunity in this review to draw attention to some recently discovered pathways of platelet activation in response to tissue damage and discuss the outcomes of each particular pathway for the most common types of CD4 T cells: Th1, Th2, Th17, and Tregs.

**Table 1:** Platelet-derived soluble factors and surface molecules that affect proliferation, differentiation, and migration of Th1, Th17, Th2, and Tregs.

Class of mediators	Platelet-derived mediators	How produced during inflammation	Effect on CD4 T cells
Cytokines	IL-1α (mouse)	Released from α-granules	Promote differentiation of Th17 cells (24, 25)
	IL-1β (human)		
	TGFβ1	Released from α-granules	Promote differentiation of Tregs; TGFβ1 in combinations with IL-6 stimulate differentiation of Th17 cells (26)

Chemokines	CXCL1	Released from α-granules	Stimulate differentiation of Th17 cells (27)
	CXCL4	Released from α-granules	Stimulate differentiation of Th17 cells (17, 28, 29)
	CCL5	Released from α-granules	Potent chemoattractant for effector memory T cells; *s*timulate Th1 and Th17 cells (28)

Neurotransmitters	Serotonin	Released from dense granules	Stimulate proliferation of CD4 T cells and differentiation of Th1 cells (17)
	Dopamine	Released from dense granules	Stimulate differentiation of Th1 and Th17 cells; inhibit Tregs (14)
	Histamine	Released from dense granules	Inhibit Tregs (30)
	Epinephrine	Released from dense granules	Indirectly promote Th2 and Th17 differentiation affecting antigen-presenting cells (31)
	GABA	Released from dense granules	Inhibit CD4 T cell proliferation (32)

Low-molecular-weight mediators	ATP	Released from dense granules	Stimulate CD4 T cell proliferation in moderate concentrations; inhibit CD4 T cell proliferation in high concentrations (33)

Lipid-derived factors	Platelet-activating factor (PAF)	Released from plasma membrane. Present on the surface of platelet-derived microparticles (PMPs)	Stimulate differentiation of Th17 cells (17)
	Thromboxane A2	Released from plasma membrane	Stimulate chemotaxis of naive CD4 T cells (34)

Other immune mediators	PMPs	Produced by activated platelets by budding process	Possibly stimulate Th17 cells *via* PAF (22); inhibit transdifferentiation of Tregs into Th17 cells *via* CD62P (35)

Adhesion molecules	CD40	Recruited to the surface from membranes of α-granules	Costimulatory signal for CD4 T cells
	CD62P	Recruited to the surface from membranes of α-granules (CD62P); constitutively expressed (αIIbβ3)	Participate in platelet–T cell aggregates; inhibit proliferation and differentiation ofTh1 and Th17 (4, 17, 21, 36)
	αIIbβ3		
	Siglecs (?)		

## Platelet Recognition of Damage-Associated Molecular Pattern (DAMP) Signals and Direct Communication with CD4 T Cells

The classic view of the initiation of inflammation and adaptive immune response holds that the sensing by innate immune cells (macrophages, dendritic cells) of pathogen or initial tissue damage is followed by the stimulation of adaptive immunity by the CD4 T cells, which in turn help to activate CD8 T cells and B cells. It is commonly agreed that innate immune cells become activated by pathogens through specific receptors such as toll-like receptors (TLRs), C-type lectin receptors (CLRs), and NOD-like receptors, but it is much less clear how these innate cells sense initial damage during sterile inflammation in the absence of infection (37). Several candidate molecules such as HMGB1 and HSPs [both of which are released by apoptotic and necrotic cells and activated platelets at the site of injury (2, 38)] have been proposed recently as activating ligands for TLRs on classic innate cells such as dendritic cells or macrophages or CD8 T cells, but many other pathways remain to be discovered (37). In this respect, platelets are well known as cells that sense initial damage to blood vessels within seconds, in the absence of any infection. Indeed, they recognize highly glycosylated structures of extracellular matrix (ECM) that become exposed to platelets when blood vessel endothelial cells become damaged. These structures include collagen, von Willebrand factor, laminin, and fibronectin, among others (7). However, the exposure of glycosylated structures of ECM to blood-derived cells is an integral part of any type tissue inflammation, when the blood vessel walls become permeable for platelets along with other blood-derived cells (e.g., leukocytes) and serum proteins (e.g., immunoglobulins) (39).

In addition to glycosylated components of ECM that are recognized by platelets in the tissues, we recently found that platelets recognize specific glycolipids (sialylated gangliosides) that are present in the detergent-resistant rigid membrane domains [also referred to as lipid rafts (40)] of postsynaptic neurons (Figure 1) and on astroglial cells that comprise the blood–brain barrier (21). Similar glycolipid-rich lipid rafts are also present on the surface of activated T cells in the area of immunological synapse, when T cells interact with antigen-presenting cells or platelets (41) (Figure 1).

Gangliosides are also present in the blood vessels (GD3), and certain organs including the brain (GM1, GD3, GT1b, and GQ), pancreas, testis, lungs, and heart are enriched with sialylated gangliosides (21). CD4 T and CD8 T cells are known to require certain gangliosides for their activation and for the assembly of lipid rafts in T-cell receptor (TCR) area during their activation (41). Particularly, it has been shown that CD4 T cells require GM1a and CD8 T cells require GM1b for their activation (42). We found that platelets recognize ganglioside GM1, GT1b, and GQ, which occur abundantly in neuronal cells, particularly in lipid rafts of postsynaptic neurons (21) (Figure 1). Most strikingly, the intravenous injection of biochemically isolated neuronal lipid rafts caused massive platelet activation and degranulation in mice, leading to anaphylactic shock (21). The exact mechanisms of this recognition of ganglioside-rich lipid rafts by platelets remains unclear, but the process evidently involves several receptors on platelets, including CD62P and possibly a number of lectins (e.g., Siglec-H, Siglec-15, CLEC-2) (21). An attractive hypothesis is that platelets can also recognize specific gangliosides on the surface of lipid rafts of activated T cells similar to those of neurons *via* specific lectins and highly glycosylated adhesion molecules such as ALCAM and/or NCAM (17) (Table 1). In support of this hypothesis, our group demonstrated that *st3gal5*-deficient mice that lacked several sialylated gangliosides such as GM1, GT1b, and GQ do not develop Th1- and Th17-mediated experimental autoimmune encephalomyelitis (EAE) (21). Two studies (one of which was by ourselves) have also recently demonstrated that mice with depleted platelets do not develop severe EAE (21, 43). Therefore, recent findings suggest that platelets are able to sense initial tissue damage-associated signals such as sialylated gangliosides in the lipid rafts within damaged tissues. As mentioned earlier, ganglioside-rich lipid rafts are present in tissue stromal and infiltrating activated immune cells such as T cells. Therefore, the evidence suggests the presence of an important link between the sensing of initial tissue damage by platelets in the absence of infection and the proliferation and differentiation of CD4 T cells at the site of injury.

## Platelet Recognition of Pathogen-Associated Molecular Pattern (PAMP) Signals

Besides being able to sense tissue damage, platelets also have a number of the receptors that directly interact with pathogens. These receptors include TLRs (TLR2, TLR3, TLR4, TLR7, TLR9), Siglecs (Siglec-H, Siglec-7, Siglec-15), and CLRs (CLEC-2). The role of TLRs, CLEC-2, and Siglecs on platelets has recently been reviewed (4). Platelets do not have MHC class II, but they express MHC class I along with the co-stimulatory molecules CD40 and CD86, and are capable of presenting antigen to CD8 T cells (44). In this review, we focus on the role of these interactions for the stimulation of CD4 T cells. Our data strongly suggest that in myelin oligodendrocyte glycoprotein (MOG)-TCR transgenic mice immunized with MOG_35-55_ peptide with complete Freund’s adjuvant (CFA), the proliferation of MOG-specific CD4 T cells was greatly decreased when platelets were depleted (17). Moreover, the production of IFNγ and IL-17 by MOG-specific CD4 T cells was also significantly decreased in immunized mice with depleted platelets (17). This indicates an important role for platelets during T cell priming *in vivo* when mice are immunized with antigen (MOG) with CFA. We believe that a similar result will be observed not only for MOG but also for most common antigens. Indeed, mice with depleted platelets displayed an elevated level of bacterial load during infection, suggesting a poor immune response against pathogens (3). Our interpretation of these results is that platelets become activated by CFA or pathogens (bacteria) and produce a number of pro-inflammatory factors that support the proliferation and differentiation of CD4 T cells toward Th1 and Th17 at the site of immunization or infection. Similar results were found for induction of EAE in mice with depleted platelets, when disease was significantly diminished (21). To induce EAE, C57BL/6 mice were immunized with MOG_35-55_/CFA, with subsequent administration of *pertussis toxin*, at day 0 and day 2. Without *pertussis toxin*, EAE could not be induced in MOG_35-55_/CFA-immunized mice. However, we managed to induce EAE in the absence of *pertussis toxin* when we administered intravenously biochemically isolated neuronal lipid rafts, which caused massive platelet activation and degranulation (21). Thus, platelet activation became an effective substitute for the action of *pertussis toxin*, which is known to activate T cells and increase the permeability of blood vessels (45). We concluded that platelet activation and degranulation were important events for the stimulation of autoimmune Th1 and Th17 cells in an EAE model.

The precise nature of the reaction by platelets to CFA and/or *pertussis toxin* remains unclear, but we believe that TLRs (e.g., TLR4) and Siglecs (e.g., Siglec-H) are involved in this process. CELC-2 could be also involved, since it has been reported that this activating receptor directly binds to HIV (4). All these receptors on platelets interact with mycobacteria in CFA. Interestingly, we found that the administration of biochemically isolated neuronal lipid rafts caused massive platelet degranulation and the complete fragmentation of the platelets into platelet-derived microparticles (PMPs), leading to thrombocytopenia (21). The formation of PMPs and the presence of thrombocytopenia is a hallmark of many infections and autoimmune diseases such as hemorrhagic fever (46) or multiple sclerosis (17). Moreover, PMPs have PAF on their surfaces (22), which have the ability to stimulate Th17 cells (17) (Table 1).

Another possibility is that massive platelet activation induced by lipid rafts acts as a systemic pro-inflammatory signal inducing the activation of innate cells through the activation of the inflammasomes and other pro-inflammatory pathways. In this hypothesis, platelets would be able to recognize DAMP and PAMP signals along with glycolipids in the lipid rafts and to directly stimulate CD4 T cells. This stimulation is important for the development of the normal immune repose to pathogens and for the development of autoimmune diseases such as multiple sclerosis. We believe that similar mechanisms of stimulation of Th1 and Th17 cells occur in several other types of Th1/17-mediated autoimmune diseases.

## Modulation of Function of Th1, Th17, and Th2 Cells by Platelets

To better understand the role of platelet-derived factors in the modulation of the functions of CD4 Cells, several research groups have performed co-culture (co-incubation) experiments on platelets with T cells. Several studies have indicated an important role for platelet-derived serotonin/5HT in the functions of T cells *in vitro* and *in vivo* (13, 47). Our own studies with polyclonally stimulated human CD4 T cells with anti-CD3/CD28 mAbs indicate that 5HT stimulates proliferation and IFNγ production by CD4 T cells and has no effect on Th17 cells (17). At the same time, platelet-derived PAF and CXCL4 stimulate the differentiation of Th17 cells (17) (Table 1). We have demonstrated that the co-incubation of platelets with CD4 T cells did not increase the number of Th2 cells. We even found a tendency for a reduction in the percentages of Th2, demonstrating that platelets skew the balance toward Th1/Th17 (17). A study with a higher number of added platelets demonstrated that platelet-derived factors CXCL4 and CCL5 also enhanced Th1 and Th17, while producing little or no effect on Th2 cells (28). Taken together, these studies suggest that 5HT, CXCL4, CCL5, and PAF play an important role in the stimulation of Th1 and T17 cells, as summarized in Table 1. Furthermore, platelets also produce CXCL1, which has been reported to stimulate Th17 cells, and may enhance or substitute for the effect of other platelet-derived factors in the stimulation of Th17 cells (Table 1). Among other platelet-derived factors, epinephrine has been shown to stimulate Th17 and Th2 cells. However, the stimulation of Th2 cells was indirect and was derived through the effect on antigen-presenting cells (Table 1). In other words, most platelet-derived factors stimulated Th1 and Th17 cells but not Th2 cells, thereby skewing the balance toward Th1 and Th17.

## Modulation of the Function of Tregs by Platelets

In our studies utilizing a physiological platelet to CD4 T cell ratio of 15 to 1 or less, we did not find stimulation of Tregs in our co-culture experiments (17). Indeed, we identified a trend for a reduction in the percentage of Tregs, much as we found for Th2 cells. However, when a high number of platelets (~100 to 1) were involved, we found that the percentage of Tregs actually increased (unpublished). Our findings were confirmed by the results of an earlier study, involving the stimulation of Tregs at a high platelet to CD4 T cell ratio (250 to 1), where the effect of the stimulation of IL-10-producing Tregs was blocked by anti-pan-TGFβ polyclonal antibodies (28). These studies suggested that platelet-derived TGFβ1 (Table 1) is capable, under certain conditions, of stimulating Tregs. A recent study has confirmed that platelets stimulate the formation of Tregs *in vivo* in the case of tumor-infiltrating CD4 T cells (48). Thus, platelet-derived TGFβ1 is able to stimulate Tregs, but only when platelets are present in high numbers. At first sight, this finding seems at odds with the fact that TGFβ1 is found in platelet α-granules in relatively high concentrations (49). The most likely explanation is that TGFβ1 is secreted by platelets into blood serum (and probably transported from serum into platelets) where this cytokine is mostly present in its biologically inactive form (50). Therefore, platelet-derived TGFβ1 is mostly inactive and should be activated by additional pathways. At the same time, platelets have the ability to secrete pro- and anti-inflammatory mediators that produce an opposite outcome for CD4 T cells (Table 1). The most critical question here is how selectively the process of secretion of anti-inflammatory (e.g., TGFβ1) vs. pro-inflammatory (e.g. CXCL4) agents is regulated *in vivo* during various pathologies. An attractive hypothesis is that various α-granules could contain different substances, which are selectively released upon specific types of stimulation. In support of this hypothesis, it has been shown that one α-granule contains fibrinogen, and another—vWF (51). Thus, there is a possibility that TGFβ1 is located in other granules than CXCL4. Future research should allow this uncertainty to be resolved.

## Formation of Platelet–T Cells Aggregates and Modulation of Function of T Cells

Besides stimulating CD4 T cells *via* soluble factors, platelets also form direct contacts with T cells, leading to their stimulation or inhibition. The role of CD40 on platelets that interact with CD40L on T cells has been shown to be important for stimulation for CD4 T cells (Table 1). However, several recent studies have demonstrated that platelets bind to CD4 T cells *via* multiple receptors, including integrins (αIIbβ3), P-selectin (CD62P), and possibly other lectins such as Siglec-H, Siglec-7, and CLEC-2 (Table 1). All of these receptors are able to bind to glycolipids and glycoproteins within lipid rafts, perturbing the interaction of CD4 T cells with antigen-presenting cells (17, 21). Several studies (including one by ourselves) have demonstrated that the formation of platelet–-CD4 T cell aggregates results in the downmodulation of proliferation and differentiation toward Th1 and Th17 (16, 17). We found that during the advanced stages of human autoimmune disease multiple sclerosis, platelets become exhausted in the content of soluble factors in their granules, but upregulated integrins, lectins, and adhesion molecules effectively bind CD4 T cells, perturbing their interaction with antigen-presenting cells or endothelial cells. This eventually inhibited T cell activation and migration to the site of tissue inflammation in a mouse EAE model (17). Similar results have also been recently found for rheumatoid arthritis patients (16). Thus, the formation of platelet-CD4 T cell aggregates mostly inhibited Th1 and Th17 cells during the resolution of tissue inflammation.

## Concluding Remarks

Recent studies have shown that platelets have two unique properties (1). They directly sense damage- and pathogen-associated molecular patterns (2). They swiftly react and directly communicate with CD4 T cells. Importantly, platelets also play a dual role in the regulation of tissue inflammation (1). During the initiation of inflammation, they produce a number of soluble substances that stimulate proliferation, differentiation, and migration Th1 and Th17 cells (2). During the resolution of inflammation, platelets inhibit proliferation and Th1 and Th17 differentiation by forming platelet–T cell aggregates. Interestingly, platelets act in many instances much like neuronal cells, and platelet-–T cell interactions represent a good model for “neuroimmunological” synapse and direct interaction between the nervous and immune systems. These findings should lead to the opening of new pathways for targeting platelets and either stimulating the immune response during infection, vaccination, or antitumor immunotherapy or inhibiting it during chronic autoimmune diseases such as rheumatoid arthritis, type I diabetes, and multiple sclerosis.

## Author Contributions

EP planned and wrote whole manuscript and prepared table and figure.

## Conflict of Interest Statement

The author declares that the research was conducted in the absence of any commercial or financial relationships that could be construed as constituting a potential conflict of interest.
